# The Rab25‐ADAMTS5 axis as a previously undescribed mechanism for sensing tumor microenvironment complexity

**DOI:** 10.1111/febs.70147

**Published:** 2025-05-23

**Authors:** François Tyckaert, Francesco Baschieri

**Affiliations:** ^1^ Institute of Pathophysiology, Biocenter Medical University of Innsbruck Austria

**Keywords:** CAF, cancer, extracellular matrix, metastasis

## Abstract

The tumor microenvironment (TME), particularly the extracellular matrix (ECM), plays a critical role in cancer progression. Focusing on ovarian cancer, Yuan *et al.* reveal an ECM‐dependent signaling axis where cancer‐associated fibroblasts (CAFs) enhance the invasiveness of cancer cells via Rab25‐driven upregulation of the protease ADAMTS5. This process is only triggered in the presence of native ECM. In turn, stimulated cancer cells favor CAF invasiveness through a mechanism that remains to be identified. These findings uncover a bidirectional crosstalk between cancer cells and CAFs and highlight the importance of context‐specific *in vitro* models to decipher ECM‐mediated tumor dynamics.

## Graphical abstract

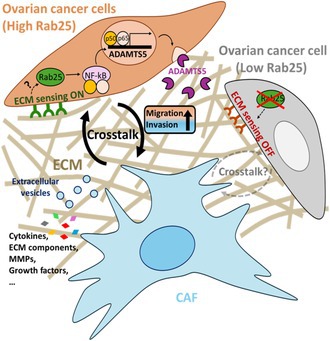

**Abbreviations**
ADAMTS: a disintegrin and metalloprotease with thrombospondin motif
CAF: cancer‐associated fibroblasts
ECM: extracellular matrix
FAP: fibroblast activation protein
LOX: lysyl oxidases
MMP: matrix metalloproteases
TGF‐β: transforming growth factor‐β
TME: tumor microenvironment
αSMA: alpha smooth muscle actin

## Introduction

One hallmark of cancer progression is the profound remodeling of the tumor microenvironment (TME), particularly of the extracellular matrix (ECM). Numerous studies have identified a correlation between stromal infiltration of tumors and disease aggressiveness [1]. Yet, despite this, the ECM is still frequently overlooked in *in vitro* studies. The ECM was viewed for long as merely a passive scaffold, difficult to manipulate experimentally, and thus not worth prioritizing over cancer cell‐intrinsic mechanisms.

However, recently, attention has shifted to the ECM and the tumor stroma as well.

Stromal cells include, among other cell types, immune cells, as well as fibroblasts and endothelial cells. While immune cells have taken center stage, thanks in part to the rise of immunotherapy, other stromal components such as fibroblasts remain underexplored. This is especially notable given that fibroblasts, and in particular cancer‐associated fibroblasts (CAFs), are often the most abundant stromal cell type in solid tumors and are strongly associated with fibrosis and worse clinical outcomes [2].

That is partly due to the fact that the study of CAFs is limited by significant experimental challenges. CAFs lack a unique marker for easy identification or isolation. Furthermore, their origin is still under debate, complicating efforts to create experimental systems in which fibroblasts are completely absent. Due to all these inherent difficulties, CAFs are commonly classified into three broad subtypes—myofibroblastic (myCAFs), inflammatory (iCAFs), and antigen‐presenting CAFs—based on the expression of markers such as TGF‐β, FAP, and αSMA, coupled with functional assays [3]. Among these subtypes, myCAFs are by far the most abundant. Their core functions include the secretion of ECM proteins and remodeling enzymes, as well as the mechanical organization of the ECM through force‐based fiber alignment.

For these reasons, myCAFs (hereafter referred to simply as CAFs) are considered central players in ECM remodeling and fibrosis.

Indeed, ECM remodeling is arguably the most defining function of all CAF subtypes, regardless of their specific classification. In fact, the ECM is constantly remodeled in health and disease, and imbalances in this remodeling can lead to the progression of pathologies.

The ECM is actively remodeled through protein secretion, crosslinking, and cleavage. While all cell types can in principle secrete ECM proteins, de facto fibroblasts are the primary architects, secreting structural ECM proteins and enzymes like lysyl oxidases (LOX) for crosslinking. Conversely, proteases such as MMPs, ADAMs, and ADAMTS can be secreted by multiple cell types, including cancer cells and fibroblasts [4].

Over 1000 genes—around 4% of the human genome—are implicated in ECM maintenance [5], reflecting the key role of the ECM in tissue homeostasis and disease. However, this complexity has hindered therapeutic translation: for instance, matrix metalloproteinases (MMP) inhibitors were initially regarded as highly promising anticancer agents, yet they failed dramatically in clinical trials [6]. To target the ECM effectively, we need a deeper, mechanistic understanding grounded in context‐specific experimental systems.

## A new role for ADAMTS5 in the ECM–cancer cell crosstalk

A disintegrin and metalloprotease with thrombospondin motif (ADAMTS) are a family of secreted zinc‐dependent metalloproteases representative of the complexity of the ECM. Their function in cancer can vary from tumor‐suppressive and antiangiogenic to tumor and metastasis‐promoting. ADAMTS5, in particular, is a secreted protease targeting proteoglycans that has demonstrated divergent roles across cancer types—protective in human hepatocellular carcinoma, melanoma, and cancers of the digestive apparatus [7], yet associated with poor prognosis in ovarian cancer [8].

Ovarian cancer remains one of the most lethal gynecological malignancies, largely because of late‐stage diagnosis when metastatic dissemination and chemoresistance have already taken hold. CAFs represent on average 50% of all the stromal cells in ovarian cancer [9], which is characterized by a highly heterogeneous microenvironment [10]. Given the links between metastatic dissemination, resistance to therapies, and CAFs abundance, it is not surprising that a synergy between CAFs and cancer cells has already been the object of attention of some studies [11]. However, even in the few instances where CAFs and cancer cells have been studied together, attention has been given to cancer cells activating or controlling CAFs, and not the other way around.

In this compelling study, Yuan *et al*. [12] focus precisely on this underexplored dynamic. Using cocultures of cancer cells and cancer‐associated fibroblasts (CAFs), they unveiled an unexpected phenotype that would have been neglected in standard monoculture experiments. The native matrix produced by CAFs, synergizing with an upregulation of the small GTPase Rab25 often present in ovarian cancer cells [13], triggered a robust increase in the secretion of ADAMTS5, driven by NF‐κB‐dependent transcriptional activation.

Functionally, ADAMTS5 proved sufficient to promote cancer cell migration and invasion across 3D matrices. Importantly, the inhibition of ADAMTS5's catalytic activity impaired pseudopodia elongation and reduced the directional persistence of migrating cancer cells—but only in the presence of the CAF‐derived matrix. On plastic, the inhibition had no effect, underscoring the critical role of ECM context in activating specific invasive programs.

Interestingly, conditioned media from Rab25‐overexpressing cells was able to trigger migration in ADAMTS5‐silenced recipient cells, confirming that secreted ADAMTS5 was sufficient to induce the invasive phenotype.

Given the established importance of cancer cell–CAF interactions in ovarian cancer metastasis [11], Yuan *et al*. examined the effect of ADAMTS5 secretion on cocultures of CAFs and cancer cells growing as spheroids in 3D environments. OVCAR3 cells, typically noninvasive in monoculture, began to invade when cocultured with CAFs in 3D matrices, the phenomenon being fully dependent on increased ADAMTS5 secretion. Even more surprising was the observation that CAFs themselves exhibited increased invasiveness when in contact with Rab25‐high cancer cells. However, the effect on CAFs was most likely not mediated by ADAMTS5 catalytic activity, as CAF monocultures stimulated with conditioned media from cells producing ADAMTS5 did not display increased invasiveness. This hints at a more complex, reciprocal crosstalk still awaiting mechanistic dissection.

## Concluding remarks

This study highlights the underappreciated influence of CAFs on cancer cell behavior and unveils a striking pathway in which CAF‐derived matrix selectively activates a Rab25–NF‐κB–ADAMTS5 axis in ovarian cancer cells. These cancer cells, in turn, seem to signal back to CAFs through an unknown mechanism, potentially reinforcing a feedback loop that accelerates invasion.

With their work, the Rainero group has demonstrated how cell biology approaches can decode the spatial and contextual specificity of cancer signaling in a way that reductionist monocultures or *in vivo* bulk analyses often cannot. But, as is often the case with cutting‐edge research, the findings raise as many questions as they answer.

Why does Rab25 trigger ADAMTS5 upregulation only on native ECM environments? In other words, how do cancer cells “read” their environment? Could ECM internalization via endocytosis play a signaling role beyond degradation [14]? Or is a second signal required for full transcriptional activation? Given Rab25's known role in trafficking α5β1 integrins and other integrins [15], it is tempting to speculate that integrin signaling contributes to such a positive feedback loop: Rab25 enhances trafficking of integrins, which bind to and remodel the ECM, leading to the activation of NF‐κB, and ultimately promoting ADAMTS5 expression.

In sum, the research elevates the TME from background to central focus, providing a methodological roadmap for rigorous cancer cell biology studies.

## Conflict of interest

The authors declare no conflict of interest.

## Author contributions

FB prepared the original draft. FT prepared the graphical abstract. Both authors reviewed and edited the manuscript and graphical abstract.
